# CD8+ T cell aging is associated with macular neovascularization area change in neovascular age-related macular degeneration: a prospective cohort study

**DOI:** 10.1186/s12886-025-04570-2

**Published:** 2026-01-10

**Authors:** Alexander Kai Thomsen, Maria Abildgaard Steffensen, Kathrine Gotfredsen, Henrik Vorum, Bent Honoré, Mogens Holst Nissen, Torben Lykke Sørensen

**Affiliations:** 1 Department of Ophthalmology, Zealand University Hospital, Roskilde, 4000 Denmark; 2 Department of Clinical Medicine, University of Copenhagen, Copenhagen, Denmark; 3 Department of Immunology and Microbiology, University of Copenhagen, Copenhagen, Denmark; 4 Department of Clinical Medicine, Aalborg University Hospital, Aalborg, Denmark; 5 Department of Ophthalmology, Aalborg University Hospital, Aalborg, Denmark; 6 Department of Biomedicine, Aarhus University, Aarhus, Denmark

**Keywords:** Age-related macular degeneration, Neovascular age-related macular degeneration, T cells, Immunosenescence, Macular neovascularization, Choroidal neovascularization, Macular neovascularization area, Treatment response, Systemic inflammation, Inflammation

## Abstract

**Background:**

Neovascular age-related macular degeneration (nAMD) is characterized by formation of macular neovascularization (MNV). The aging immune system plays an important role in nAMD pathogenesis. Loss of the costimulatory markers CD27 and CD28 on T cells and increased T cell differentiation is associated with immunosenescence and proinflammatory T cell activation. In this study we investigate the association between MNV area change following anti-VEGF treatment and the aging T cell profile in nAMD patients.

**Methods:**

This prospective cohort study included treatment-naïve nAMD patients. Participants were examined with optical coherence tomography angiography at time of diagnosis and following loading dose to assess the MNV area change. A blood sample was analyzed for circulating aging T cell profile with flow cytometry for the costimulatory markers CD27, CD28, and CD56, as well as T cell differentiation (naïve, central memory, and effector memory) of CD4+ and CD8+ T cells.

**Results:**

54 eyes of 54 patients were included. A significant association was found between a reduction of MNV area and a reduction of the CD8+CD27- T cell proportion (β, 0.71; 95% CI, 0.17 to 1.26; *P* = 0.035), as well as CD8+CD28- T cell proportion (β, 0.72; 95% CI, 0.20 to 1.23; *P* = 0.035). A non-significant negative trend was observed between MNV area change and CD8 + naïve T cells (*P* = 0.099).

**Conclusion:**

Our results suggest that a less advanced aging T cell profile characterized by lower levels of CD8+CD27- and CD8+CD28- T cells is associated with a greater reduction of MNV area following anti-VEGF treatment in treatment-naïve nAMD patients.

**Supplementary Information:**

The online version contains supplementary material available at 10.1186/s12886-025-04570-2.

## Introduction

A leading cause of irreversible vision loss and legal blindness in the elderly is age-related macular degeneration (AMD). Neovascular AMD (nAMD) is a late stage characterized by the formation of a macular neovascularization (MNV), which can cause leakage of intra- and subretinal fluid, and hemorrhages [1]. A key driver of MNVs is vascular endothelial growth factor (VEGF), which can be inhibited in the treatment of nAMD through intravitreal anti-VEGF injections. However, treatment response varies significantly between patients, and a considerable proportion of patients experience deterioration of visual function despite treatment [2]. Alternative treatment options are needed for these patients [3].

Changes in MNV characteristics, as quantified using optical coherence tomography angiography (OCTA), have been suggested as potential markers of treatment response in nAMD patients [4]. Since MNVs cause the anatomical changes in the retina that lead to visual impairment, the MNV itself may be a key pathological marker to target [5]. OCTA is a non-invasive imaging modality capable of detecting and visualizing retinal and choroidal blood vessels, including MNVs [6]. Previous studies have demonstrated that the MNV area, as measured by OCTA, tends to decrease following anti-VEGF treatment. However, the extent of MNV area reduction varies significantly between individual patients [4, 7, 8]. Furthermore, the greatest linear dimension (GLD) of the MNVs has been shown to decrease with treatment [9].

**Fig. 1:**
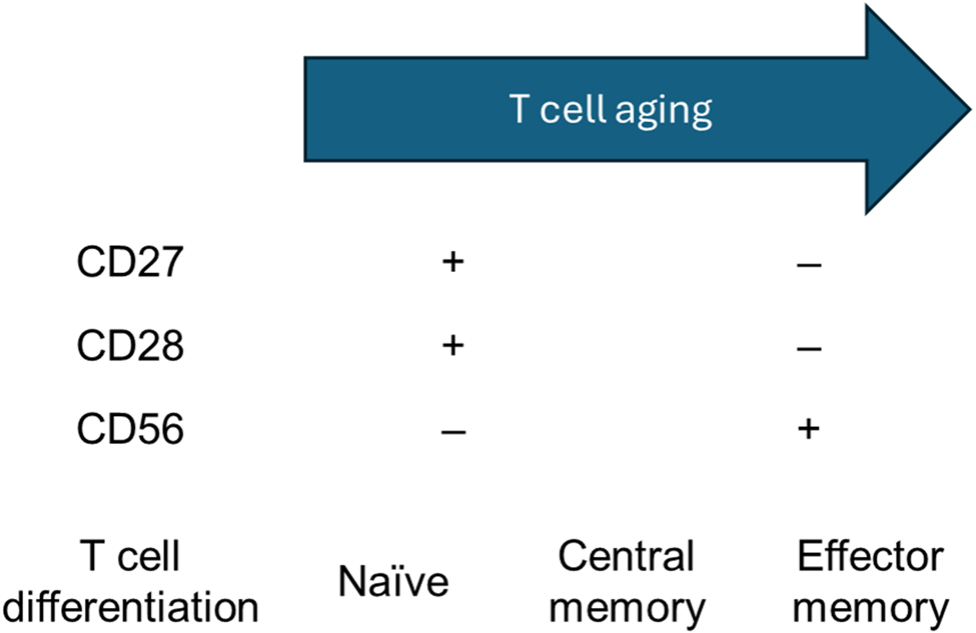
Aging T cells are characterized by downregulation of the costimulatory markers CD27 and CD28, as well as upregulation of CD56. Furthermore, more advanced T cell differentiation occurs with naïve T cells differentiating to central memory and effector memory T cells

The pathogenesis of AMD is not fully understood, as AMD is a multifactorial disease [10]. Age is the most important risk factor for the development of AMD, and emerging evidence suggests that systemic inflammation might play an important role [11]. The immune system undergoes dynamic age-related changes known as immunosenescence [12]. This can lead to a state of low-grade chronic inflammation, termed inflammaging, and is associated with AMD stage [13]. Specifically, age-related differentiation of T cells has been found to be associated with AMD and treatment response in nAMD patients [14, 15]. Aging T cells are characterized by the loss of the costimulatory markers CD27 and CD28 [16], as well as the upregulation of CD56 [17]. The total number of T cells decreases with age [18]; however, a larger proportion of T cells will differentiate from naïve to central memory and effector memory T cells (Fig. 1) [19]. These changes and dysregulation can increase systemic inflammation, which can cause tissue damage and angiogenesis [20].

Based on these prior findings, we investigate whether MNV area change following treatment quantified on OCTA is associated with the aging T cell profile in treatment-naïve nAMD patients. This might contribute to the understanding of T cell aging in the pathogenesis of nAMD and the potential role of targeting aging T cells as a treatment in nAMD. A sub-analysis investigating the association between GLD and aging T cell profile was also performed.

## Methods

### Study design and participants

The Danish Neovascular Age-Related Macular Degeneration and Treatment Response (DANEART) study is a prospective cohort study investigating immune profiles in AMD patients. This single-center study is conducted at the Department of Ophthalmology, Zealand University Hospital, Denmark, and has received approval from the Regional Committee of Ethics in Research for the Region of Zealand, Denmark (journal no. SJ-768). All procedures are conducted in compliance with the Declaration of Helsinki. Informed consent, both verbal and written, was obtained from all participants prior to their enrollment.

The DANEART study design and participants have been described in detail previously [21]. In summary, this study included treatment-naïve patients with nAMD. The eye with nAMD was chosen as study eye, and in case of bilateral nAMD, the right eye was chosen. Exclusion criteria were age younger than 60 years, active infections and cancer, inflammatory and autoimmune diseases, use of immunomodulating treatment, active smoking, plasma C-reactive protein (CRP) >15 mg/L, previous anti-VEGF treatment, and other vision-affecting diseases than nAMD.

Participants underwent a baseline examination consisting of assessment of best-corrected visual acuity (BCVA), slit-lamp biomicroscopy, color fundus photography, spectral-domain optical coherence tomography (OCT), and OCT angiography (OCTA). Patients were treated with a loading phase consisting of three intravitreal anti-VEGF injections (aflibercept, 2 mg) with a month’s interval. Patients were examined a month after the last injection to determine initial treatment response.

### Macular neovascularization area

**Fig. 2:**
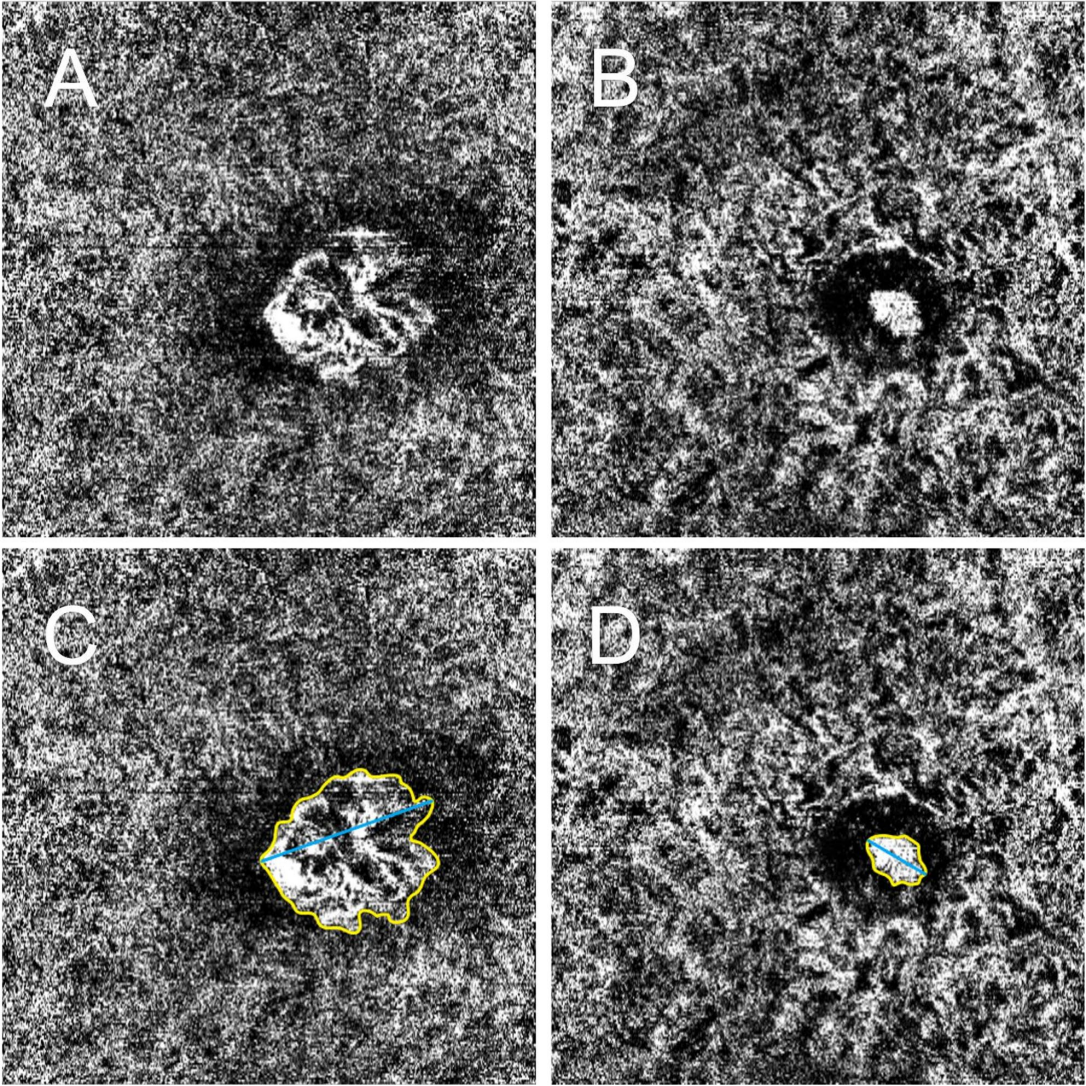
Example of OCTA imaging of macular neovascularization (MNV) before and after treatment. (**A**) MNV at baseline. (**B**) MNV following loading dose treatment. (**C**) Baseline image with MNV outlined to measure area (yellow contour) and greatest linear dimension (GLD, blue line). (**D**) Post-treatment image with MNV outlined to measure area (yellow contour) and GLD (blue line)

The MNV area was evaluated on OCTA scans. OCTA scans were performed on the Heidelberg Spectralis (Heidelberg Engineering, Heidelberg, Germany) with a 10° × 10° scan field consisting of 256 B-scans, 512 A-scans per B-scan, 7 averaged frames in automatic real time. The best slab to visualize the MNV en face on OCTA was found in HEYEX (Heidelberg Engineering, vesion 1.10.4.0) and exported to ImageJ (National Institute of Health, Bethesda, Maryland, USA). The MNV was measured manually by delineating the outer boundary and the lesion area was calculated automatically. The GLD was measured by delineating the longest width of the lesion (Fig. 2).

The MNV area and GLD were measured at baseline and following loading dose, and the changes were found by subtracting the follow-up measurement by the baseline measurement.

### Flow cytometry

**Fig. 3:**
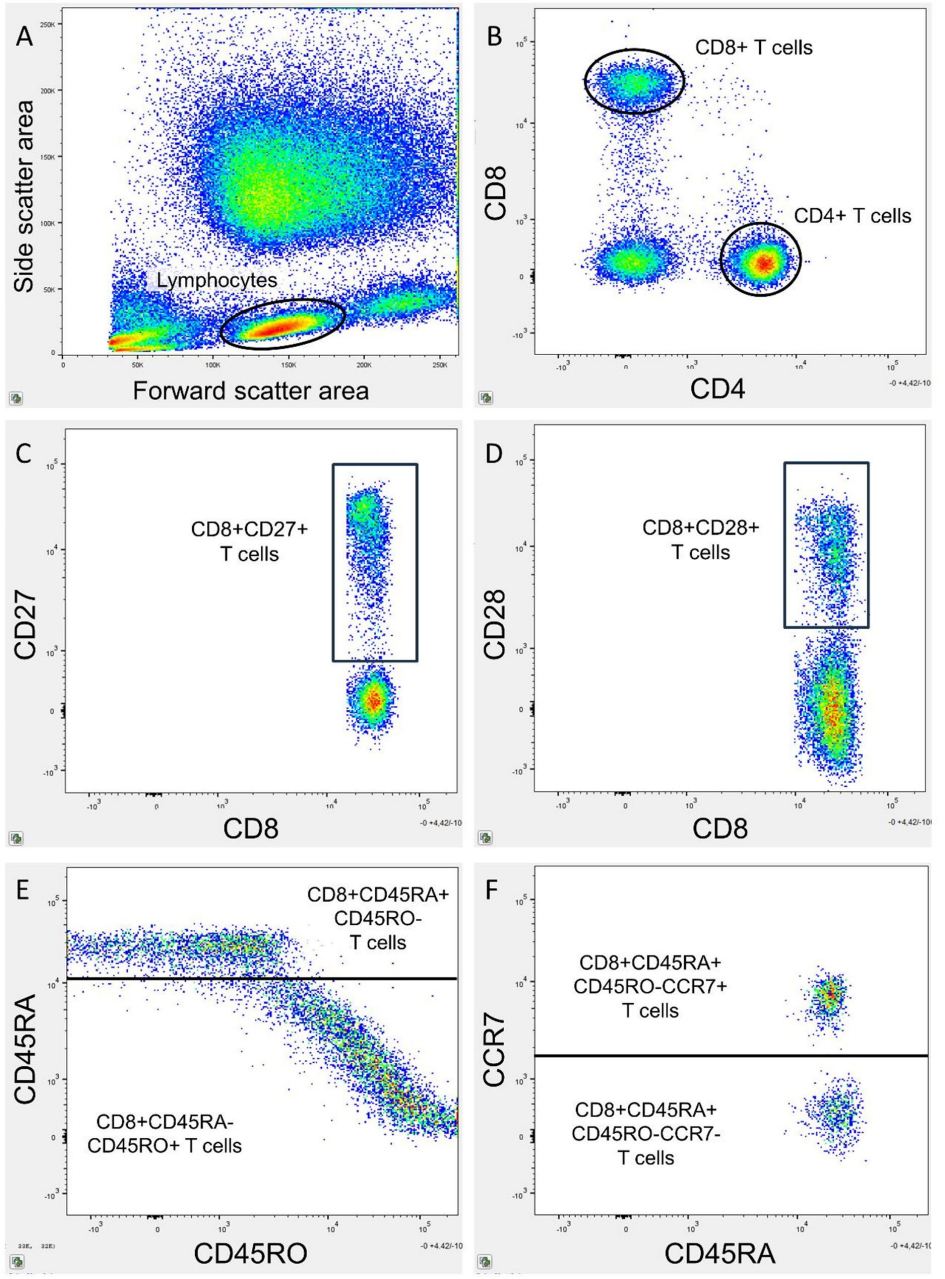
Example of flow cytometry gating strategy. Gating was performed with Boolean sequencing dividing the cell population in increasingly specific subgroups. (**A**) Lymphocytes were identified on a forward-side-scatter plot. (**B**) Lymphocytes were gated for CD4 and CD8 to identify CD4+ and CD8+ T cells. These T cells were gated for age-related surface markers, such as (**C**) CD27 and (**D**) CD28 on CD8+ T cells. (**E**) The expression of CD45RA and CD45RO was determined on T cells, in this example on CD8+ T cells. (**F**) The expression of CCR7 was determined on CD45RA+CD45RO- and CD45RA-CD45RO+ T cells, in this example on CD8+CD45RA+CD45RO- T cells

T cell differentiation profile was determined with flow cytometry. Details on the flow cytometry protocol have been described previously [22]. In brief, peripheral blood was sampled in ethylenediamine tetraacetic acid (EDTA) coated tubes. Leukocytes were isolated and stained with monoclonal fluorescent antibodies binding CD4, CD8, CD27, CD28, CD56, CD45RO, CD45RA, and C-C motif receptor 7 (CCR7). Details on flow cytometry preparations and monoclonal fluorescent antibodies can be found in Additional file 1. Flow cytometry was performed on the BD FACS Canto II flow cytometer (BD Bioscience, San Jose, CA, USA) with a gating size of 100,000 cells. Flow cytometric analyses were performed on FlowJo analytical software (Tree Star, Ashland, OR, USA, v.10.10.0). Gating was performed with Boolean sequences by identifying singlet lymphocytes. These lymphocytes were then gated for CD4 and CD8 to determine CD4+ and CD8+ T cells. T cells were gated for the costimulatory markers CD27, CD28, and CD56. T cells were also gated for CD45RA, CD45RO and CCR7 to determine T cell differentiation, and classified as naïve (CD45RA+CD45RO-CCR7+), central memory (CD45RA-CD45RO+CCR7+), and effector memory (CD45RA-CD45RO+CCR7-) [15, 23, 24] (Fig. 3).

### Statistics

Statistical analysis was performed using R software version 4.2.3 (R Foundation for Statistical Computing, Vienna, Austria). Normality was assessed using histograms and the Shapiro-Wilk test. Demographic data are presented as the mean and standard deviation (SD). T cell markers were logarithmically transformed to normalize the data. Associations between MNV area and GLD change and the T cell aging profile were tested using analysis of covariance (ANCOVA), adjusting for age, as age-related T cell differentiation is well documented [23], as well as sex and baseline BCVA. Results are presented as the B coefficient (β) and 95% confidence interval (95% CI). Baseline and post-loading dose MNV areas and GLDs are presented as the median and interquartile range (IQR). Paired t-tests were used to analyze the difference between baseline and follow-up MNV areas and GLDs. For this analysis, logarithmic transformation was applied to normalize the data. The results of the t-tests are presented as the mean difference and 95% CI in percentages. *P* values were adjusted for multiple testing using the false discovery rate (FDR) method. A *P* value < 0.05 was considered statistically significant.

## Results

### Study population

54 eyes of 54 nAMD patients were included. Mean age was 77.9 (SD, 7.2) years, 33 (61%) were female, and mean baseline BCVA of the study eye was 60.1 (SD, 15.4) Early Treatment of Diabetic Retinopathy Study (ETDRS) letters. Median baseline MNV area was 1.38 mm^2^ (IQR, 2.12) and post-loading dose MNV area was 1.19 mm^2^ (IQR, 1.78) for all participants. MNV area significantly decreased following loading dose for individual patients (mean difference, -44.1%; 95% CI, -156.9% to -66.1%; *P* = 0.0022). Median baseline and post-loading dose GLD were 1.78 (IQR, 1.02) and 1.71 (IQR, 1.57), respectively, which was not statistically significantly different (*P* = 0.18).

### MNV area change and T cell differentiation

MNV area reduction was significantly associated with a reduced proportion of CD8+CD27- T cells (β, 0.71; 95% CI, 0.17 to 1.26; *P* = 0.035). MNV area reduction was also significantly associated with a reduced proportion of CD8+CD28- T cells (β, 0.72; 95% CI, 0.20 to 1.23; *P* = 0.035).

There were no significant associations between CD8+CD56+ T cells or any costimulatory markers on CD4+ T cells. There was a non-significant tendency between MNV area reduction and increased proportions of CD4+CD56+ T cells (β, -1.52; 95% CI, -3.26 to 0.22; *P* = 0.17) (Table 1).

There were no significant associations between MNV area change and CD4+ or CD8+ T cell differentiation. There was a tendency of an association between MNV area reduction and increased proportions of CD8+ naïve T cells, however not significant after FDR corrections (β, -0.16; 95% CI, -0.29 to -0.03; *P* = 0.060) (Table 1).

There were no significant associations between GLD change and aging T cell profile (Additional file 2).

**Table 1:** Association between MNV area change and T cell costimulatory markers and differentiation

	B coefficient (95% CI)	*P* value*
**T cell costimulatory markers**		
CD4+ T cells		
CD4+CD27-	0.60 (-0.65 to 1.86)	0.41
CD4+CD28-	1.41 (-0.62 to 3.44)	0.25
CD4+CD56+	-1.52 (-3.26 to 0.22)	0.17
CD8+ T cells		
CD8+CD27-	0.71 (0.17 to 1.26)	**0.035**
CD8+CD28-	0.72 (0.20 to 1.23)	**0.035**
CD8+CD56+	0.17 (-0.31 to 0.66)	0.48
**T cell differentiation**		
CD4+ T cells		
CD4+ Naïve	-0.25 (-0.58 to 0.18)	0.49
CD4+ Central memory	0.23 (-0.62 to 1.08)	0.71
CD4+ Effector memory	0.26 (-0.27 to 0.80)	0.49
CD8+ T cells		
CD8+ Naïve	-0.16 (-0.29 to -0.03)	0.099
CD8+ Central memory	-0.07 (-0.59 to 0.45)	0.78
CD8+ Effector memory	0.29 (-0.23 to 0.81)	0.49

Bold values indicate statistical significance

*ANCOVA adjusted for age, sex and baseline BCVA with false discovery rate corrections

## Discussion

In this prospective study, we found a significant positive association between MNV area change and the baseline proportion of circulating CD8+CD27- T cells and CD8+CD28- T cells in nAMD patients. These results suggest that MNV area decreases with lower proportions of aging CD8+ T cells following anti-VEGF treatment. This further supports the theory that a proinflammatory and aging systemic immune system is involved in the pathogenesis of nAMD [13]. Previous studies have found increased levels of circulating CD28- T cells in AMD patients compared to healthy controls [14]. Our group has also in a recent study shown that nAMD patients seem to have an increased proportion of CD8+CD27- and CD8+CD28- circulating T cells compared to healthy controls [22]. In the same study, we investigated the treatment response based on central retinal thickness and retinal fluid on OCT in nAMD patients, which showed a tendency of increased CD8+CD27- T cells in poor responders compared to good responders following the loading phase. The differentiation of CD8+ T cells is particularly crucial, as this subset is most impacted by aging [19]. Aging CD8+ T cells are highly inflammatory and might damage healthy tissue [25]. Thus, aging T cells losing the expression of the costimulatory markers CD27 and CD28 may play a role in the development of AMD as well as the MNV treatment response of nAMD. Aging T cells accumulate with chronological aging and are induced by environmental factors such as oxidative stress and infections [26, 27]. Genetics are also associated with T cell aging, which contributes to the individual differences in same-aged people [28]. Likewise, these are important risk factors in AMD development [29], and part of the pathogenesis might lie in the aging T cell profile.

Inflammaging is associated with multiple age-related diseases such as Alzheimer’s disease, cancer, cardiovascular diseases, and type 2 diabetes [30–34]. The low-grade chronic inflammation causes minor, but constant tissue disruption, which eventually can cause chronic diseases [35]. Multiple inflammatory pathways have been observed to be dysregulated in AMD, including the complement system [21], chemokine system [36, 37], monocytes [38], neutrophils [39], oxidative stress [40], and cytokines [41]. These systems constitute pathways in both the innate and adaptive system but are highly interactive and are also associated with T cell aging profile and differentiation [42]. This includes interferon-gamma, which is a proinflammatory cytokine that stimulates the cytotoxic and inflammatory effects of T cells [43]. Activated T cells also secrete proinflammatory cytokines, which recruit and activate further inflammatory cells [44]. CD8+ T cells have also been found in the choroid of AMD eyes [45]. Thus, we speculate that the increased systemic proportions of aged CD8+ T cells might manifest locally in the eye, as a proinflammatory environment can stimulate ocular angiogenesis, which is a key factor in nAMD [46].

Few studies have investigated the function of T cells in MNV formation; however reduced proportions of circulating CD4+ and CD8+ T cells expressing chemokine receptor CXCR3 have been found in nAMD patients. As this receptor inhibits angiogenesis, these reduced proportions might contribute to MNV formation and growth [47, 48]. Studies investigating murine models have also found a protective effect of IL-17 A-producing γδ T cells in laser induced MNVs [49], as well as an activation caused by Sema4D-PlexinB1, which is activated by smoking [50], and inhibition of regulatory T cell derived exomes [51].

Proinflammatory cytokines which can be produced by differentiated CD8+ T cells [52], have also shown to enhance the secretion of VEGF, which can lead to MNV formation [41]. A previous study showed interleukin (IL)-1 receptor antagonist treatment, which inhibits the proinflammatory IL-1β could suppress MNVs in a rodent model [53]. Tumor necrosis factor-α has also been shown to promote MNV formation via upregulation of VEGF secretion [54]. Macrophages might also be involved in the formation of MNVs [55], as well as neutrophils [56]. The neutrophil-to-lymphocyte ratio, which is a well described indicator of inflammation [57], has been related to the MNV lesion size evaluated with fluorescein angiography [58].

We find a non-significant negative trend between MNV area change and CD8+ naïve T cells. This suggests that MNV area decreases with increased CD8+ naïve T cells following anti-VEGF treatment. This corresponds with the theory that a less differentiated T cell profile is associated with lower proportions of cytotoxic T cells, and thus less inflammation [19]. Differentiated memory CD8+ T cells possess cytotoxic properties and dysregulation can cause retinal degeneration [59, 60]. Increased proportions of undifferentiated CD8+ T cells might thus be protective, and MNVs more responsive to anti-VEGF treatment.

It is also worth noting that patients in this study were treated with aflibercept, which inhibits VEGF as well as placental growth factor (PGF), a member of the VEGF family [61]. Elevated PGF levels have been observed in the aqueous humor [62] and retina [63] of nAMD patients. Murine models have also shown increased PGF levels in eyes with laser-induced MNVs. Furthermore, anti-PGF treatment inhibits MNV growth in such models, which might be caused by suppression of mononuclear phagocytes and reduction of the proinflammatory cytokines IL-1β and IL-6 [61]. Given that these factors highly stimulate differentiation of CD8+ T cells [22], patients with decreased levels of CD8+CD27- T cells and CD8+CD28- T cells might have a more pronounced reduction in MVN area caused by the PGF inhibition in addition to the VEGF inhibition.

We find that MNV area measured on OCTA significantly decreased after anti-VEGF treatment. This is similar to previous studies investigating MNV area change in treatment-naïve nAMD patients [4, 7]. One study also reporting MNV area change in percentages found a reduction of 50% after three months of treatment [8], similar to the 44.1% decrease in this study. Previous studies also found MNV area to be predictive of clinical treatment response, in the form of persistent retinal fluid in nAMD patients [64]. Other studies did not replicate this but found other MNV characteristics such as vessel density [65] and greatest vascular caliber [66] to be predictive of treatment response. We did not find a significant decrease in GLD following loading dose, contradictory to previous findings [9], which we speculate might be caused by different imaging protocols and treatment regimens.

A significant aim of treatment for nAMD patients is retinal function, including BCVA, which is associated with MNV change [7]. Boscia et al. showed a reduction of MNV area and improvement of BCVA in diabetic retinopathy patients [67], and Sacconi et al. reported no significant change in BCVA and MNV area in patients with geographic atrophy and MNV [68]. It should also be considered that the association between aging CD8+ T cells and reduced anti-VEGF response might reflect a broader systemic vascular interaction. Kremers et al. demonstrated that electroretinographic (ERG) responses to periodic stimuli correlate with visual perception in primates, suggesting that dynamic functional testing could complement structural metrics such as MNV in evaluating treatment outcomes in retinal diseases [69]. Furthermore, Tsay et al. reported that pre-stimulus bioelectrical activity under different light adaptations influences ERG responses, implying that background retinal physiology may modulate therapeutic effects [70]. Future studies might investigate how these factors are associated with aging T cell profile. Furthermore, future studies are needed to investigate the mechanism of aging CD8+ T cells and MNV formation in the retina.

Limitations of this study include the observational design, which prevents definitive conclusions about causality. We did not correct for other structural factors that might affect the MNV area change, such as MNV type. This might be a potential confounder, which has previously been described [71, 72], and it would be valuable for future studies to consider correcting for this factor [67]. The association between MNV area and leakage is not fully understood, and the clinical response might depend on other factors of the MNV than can be identified on OCTA. The lack of functional outcomes is also a limitation, which could have contributed to the clinical relevance of aging T cells. The presence of aging T cells in the retina and choroid was not investigated, and we can only speculate that the systemic alterations manifest locally. Furthermore, additional parameters of systemic inflammation were not adjusted for. As this was a clinical study, we only included patients following the Danish national guidelines treating with aflibercept. Future studies could also investigate the effects of treatment with other anti-VEGF agents, as these might show different immune interactions. The association between other MNV characteristics, such as vessel density, might also be relevant to investigate in future studies. The statistically non-significant tendencies should be considered exploratory and interpreted likewise, as the study may have been underpowered to detect these subtle effects.

In conclusion, we found that increased proportions of CD8+CD27- and CD8+CD28- T cells were associated with MNV area changes following initial anti-VEGF treatment in nAMD patients. This suggests that an aging T cell profile might be involved in the pathophysiology of MNV formation. Future studies are needed to investigate the potential role of aging T cells as a treatment target in nAMD patients.

## Supplementary Information

Below is the link to the electronic supplementary material.

Supplementary Material 1

Supplementary Material 2

## Data Availability

The data are available from the corresponding author upon reasonable request.

## Abbreviations

AMD: age-related macular degeneration
BCVA: best corrected visual acuity
CD: cluster of differentiation
CCR: C-C motif chemokine receptor
CI: confidence interval
CRP: C-reactive protein
ETDRS: Early Treatment of Diabetic Retinopathy Study
FDR: false discovery rate
IL: interleukin
IQR: interquartile range
MNV: macular neovascularization
nAMD: neovascular age-related macular degeneration
OCT: optical coherence tomography
OCTA: optical coherence tomography angiography
PGF: placental growth factor
SD: standard deviation
VEGF: vascular endothelial growth factor
