# Supplementary material for: CD8+ T cell aging is associated with macular neovascularization area change in neovascular age-related macular degeneration: a prospective cohort study
Source: BMC Ophthalmol. 2026 Jan 10;26:66. doi: 10.1186/s12886-025-04570-2 (PMC12882413; doi:10.1186/s12886-025-04570-2)
Supplement: Supplementary file 1 — Supplementary Material 1 [file 12886_2025_4570_MOESM1_ESM.docx]

**Additional file 1**

| Flow cytometry preparations were started within 4 hours following phlebotomy. |
| --- |
| The Sysmex KX-21NTM (Sysmex Corporation, Kobe, Japan) was used to measure the leukocyte count to calculate blood volume, ensuring 1.0 × 10^6^ leukocytes for analysis. |
| A 1% erythrocyte lysis buffer was added to the sample to lyse erythrocytes. |
| The blood sample was stored at room temperature in the dark for 10 minutes. |
| Cells were washed three times by adding BD FACS Flow isotonic buffer to the sample, centrifuging at 500g for 5 minutes, followed by decantation of the supernatant. |
| The isolated leukocytes were resuspended in isotonic buffer. |
| Monoclonal fluorescent antibodies fluorescein isothiocyanate (FITC) CD4 (Abcam, cat.no. ab59474), peridinin-chlorophyll-protein (PerCP) CD8 (Biolegend, cat.no. 300922), phycoerythrin (PE) CD27 (Biolegend, cat.no. 356406), allophycocyanin (APC) CD28 (Biolegend, cat.no. 302912), and allophycocyanin-cyanine 7 (APC-Cy7) CD56 (Biolegend, cat.no. 300926), Brilliant Violet 510 CCR7 (Biolegend, cat.no. 353232), Pacific Blue CD45RA (Biolegend, cat.no. 304123), and phycoerythrin-cyanine7 (PE/Cy7) CD45RO (Biolegend, cat.no. 304230), were added to the sample. |
| The sample was incubated at room temperature in the dark for 20 minutes. |
| The stained leukocytes were washed and resuspended in isotonic buffer to remove excess fluorescent antibodies. |
| The sample was analyzed with the BD FACS Canto II flow cytometer (BD Bioscience, San Jose, CA, USA) with a gating size of 100.000 singlet cells per sample. |

**Supplementary Table 1**. Flow Cytometry Protocol.
