# Supplementary material for: CD8+ T cell aging is associated with macular neovascularization area change in neovascular age-related macular degeneration: a prospective cohort study
Source: BMC Ophthalmol. 2026 Jan 10;26:66. doi: 10.1186/s12886-025-04570-2 (PMC12882413; doi:10.1186/s12886-025-04570-2)
Supplement: Supplementary file 2 — Supplementary Material 2 [file 12886_2025_4570_MOESM2_ESM.docx]

**Additional file 2**

|  | **B coefficient (95% CI)** | ***P* value*** |
| --- | --- | --- |
| **T cell costimulatory markers** |  |  |
| CD4+ T cells |  |  |
| CD4+CD27- | -0.24 (-0.99 to 0.51) | 0.53 |
| CD4+CD28- | -0.02 (-1.26 to 1.22) | 0.98 |
| CD4+CD56+ | -0.35 (-1.42 to 0.72) | 0.51 |
| CD8+ T cells |  |  |
| CD8+CD27- | 0.21 (-0.14 to 0.55) | 0.23 |
| CD8+CD28- | 0.29 (-0.34 to 0.61) | 0.078 |
| CD8+CD56+ | 0.12 (-0.16 to 0.41) | 0.39 |
| **T cell differentiation** |  |  |
| CD4+ T cells |  |  |
| CD4+ Naïve | 0.05 (-0.20 to 0.31) | 0.67 |
| CD4+ Central memory | -0.11 (-0.62 to 0.40) | 0.67 |
| CD4+ Effector memory | -0.02 (-0.35 to 0.30) | 0.89 |
| CD8+ T cells |  |  |
| CD8+ Naïve | -0.07 (-0.14 to 0.01) | 0.079 |
| CD8+ Central memory | 0.02 (-0.29 to 0.33) | 0.89 |
| CD8+ Effector memory | 0.21 (-0.10 to 0.52) | 0.18 |
| Bold values indicate statistical significance.  *ANCOVA adjusted for age, sex and baseline BCVA. | | |

**Supplementary table 2**. Association between MNV greatest linear dimension change and T cell costimulatory markers and differentiation.
